# Chinese C allele carriers of the *ERCC5* rs1047768 polymorphism are more sensitive to platinum-based chemotherapy: a meta-analysis

**DOI:** 10.18632/oncotarget.18981

**Published:** 2017-07-04

**Authors:** Meizhen Xu, Yina Liu, Dan Li, Xuelin Wang, Shuang Liang, Gaochuan Zhang, Xiaoqin Yang

**Affiliations:** ^1^ School of Biology and Basic Medical Sciences, Soochow University, Suzhou, China; ^2^ Clinical Translational Research Center, Shanghai Pulmonary Hospital, Tongji University, Shanghai, China; ^3^ School of Life Science and Technology, Tongji University, Shanghai, China; ^4^ Yushan Town, Kunshan, China; ^5^ Scotch Plains, New Jersey, USA; ^6^ Present address: Jiayin BioTechnology Co., Ltd., Shanghai, China

**Keywords:** ERCC5, polymorphism, cancer, meta-analysis, platinum-based chemotherapy

## Abstract

It is suspected that *ERCC5* rs1047768 and rs17655 polymorphisms influence the response to platinum-based chemotherapy. This meta-analysis was performed to summarize the scattered evidence regarding the association between these two polymorphisms and sensitivity to platinum-based treatment. Thirteen studies were included after a comprehensive literature search. The pooled odds ratios and 95% confidence intervals suggested that the C allele of the *ERCC5* rs1047768 polymorphism is associated with elevated sensitivity to platinating agents, especially for Chinese patients. However, no difference among rs17655 genotypes could be detected.

## INTRODUCTION

Platinum-based chemotherapies are regarded as the most efficacious cancer treatment option. However, individual responses to these therapies greatly differ among patients due to multifactorial (intrinsic and acquired) resistance caused by genetic and epigenetic differences in sensitivity [1]. Platinum-based chemotherapy exerts its antitumor effect by suppressing DNA replication through the formation of platinum-DNA adducts, which would be recognized and remedied by cellular DNA repair mechanisms [2–4]. Genetic variation of those DNA repair systems could be one of the indicators of sensitivity to platinum-based chemotherapy [5–7]. Ideally, identifying cancer patients sensitive to platinum agents before chemotherapy could promote individualized cancer treatment, and may improve survival.

Excision Repair Cross-Complementation Group 5 gene (ERCC5, 13q33.1) encodes a critical DNA repair enzyme involved in nucleotide excision repair (NER) function. Scattered evidences indicate the variants of the *ERCC5* rs1047768 (T335C, His46His) polymorphism could yield differing survival outcomes, despite being a coding-synonymous polymorphism [8, 9]. The correlation may be explained by its possible linkage with other non-synonymous polymorphisms, or its subtle influence on enzyme conformation leading to the change of its activity or substrate specificity [10]. Several studies reported that the C allele of the *ERCC5* rs17655 (G3507C, Asp1104His) polymorphism was associated with an impaired prognosis [11, 12]. The rs17655 polymorphism sits in the C-terminal region, which is required for ERCC5’s interaction with Transcription Factor II H (TFIIH) complex in human DNA NER pathway [13]. The amino-acid substitution caused by this polymorphism may lead to differential interacting affinities, and consequently impact NER efficiency [14]. The effect of these two *ERCC5* polymorphisms on the efficacy of platinum-based chemotherapy should be quantitatively evaluated.

Many studies have tried to assess the association between the two *ERCC5* polymorphisms and the sensitivity of platinum-based therapy [9, 15–26]. However, there is not a clear consensus due to many confounding factors including cancer types, ethnicity difference, inconsistent response evaluation criteria for chemotherapy, and limited sample size in every single study. Our meta-analysis examined the influence of the *ERCC5* rs1047768 and rs17655 polymorphisms on the sensitivity to platinum-based chemotherapy in cancers.

## RESULTS

### Study characteristics

**Figure 1:**
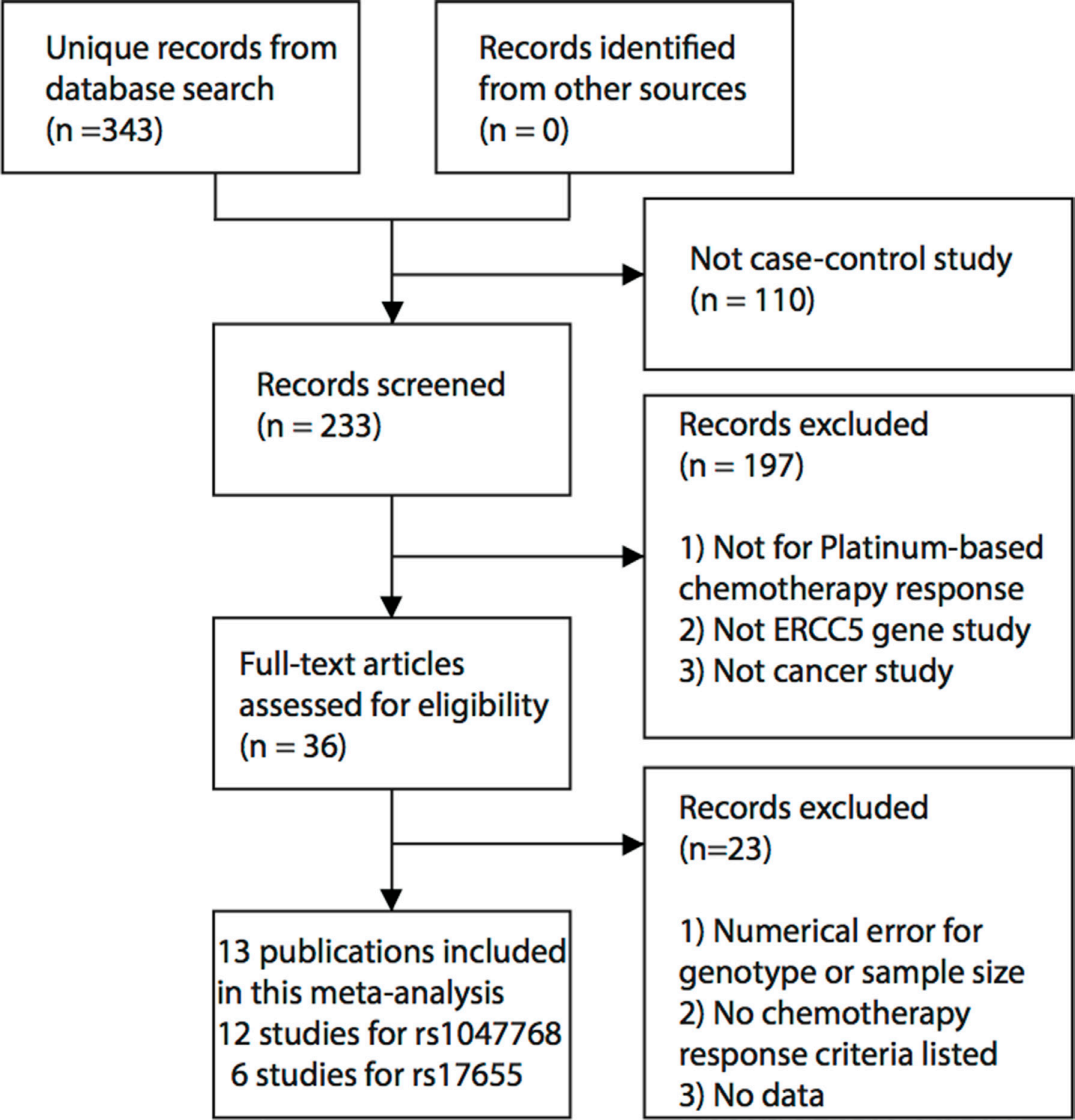
Workflow for the literature selection process of this meta-analysis

Our comprehensive literature search captured eligible 13 manuscripts [9, 15–26]. These studies were published between 2007 and 2016. One non-small cell lung cancer (NSCLC) study was split into two studies because the patient populations of the Tumor-Node-Metastasis (TNM) stage III and IV were reported separately [19]. Overall, 12 studies involving 1,506 cancer patients were eligible for the meta-analysis for the *ERCC5* rs1047768 polymorphism. For the rs17655 polymorphism, six studies with 973 samples met our predefined eligibility criteria. The selection process for eligible studies can be seen in Figure 1, and Table 1 presents an overview of the selected studies. These included studies covered NSCLC, colorectal cancer, gastric cancer, and ovarian cancer.

**Table 1:** Major characteristics of involved studies for the association between the *ERCC5* rs1047768 and rs17655 polymorphisms and sensitivity to platinum-based chemotherapy in this meta-analysis

Author	Year	Country	Reference ID	Cancer	Response Criteria	good Responder	poor Responder	Genotype Model
rs1047768								
Feng	2009	China	15	non-small cell lung cancer	WHO	30	85	CC,CT,TT
Hu	2015	China	24	epithelial ovarian cancer	PFS based	88	65	CC+CT,TT
Huang	2015	China	26	rectal cancer	RECIST	27	18	CC,CT,TT
Jia	2011	China	25	non-small cell lung cancer	RECIST	31	58	CC,CT+TT
Lv	2012	China	21	non-small cell lung cancer	RECIST	26	59	CC,CT+TT
Monzo	2007	Spain	16	colorectal cancer	RECIST	22	20	CC,CT+TT
Qin	2016	China	17	rectal cancer	TRG	81	90	CC,CT,TT
Song	2010	China	22	gastric cancer	RECIST	25	67	CC,CT,TT
Sullivan	2014	Spain	19	non-small cell lung cancer^†^	RECIST	58	16	CC,CT,TT
Sullivan	2014	Spain	19	non-small cell lung cancer^‡^	RECIST	31	56	CC,CT,TT
Zhang	2013	China	9	non-small cell lung cancer	EORTC	137	314	CC,CT,TT
Zhang	2012	China	23	ovarian cancer	NCCN	66	36	CC,CT,TT
rs17655								
Hu	2015	China	24	epithelial ovarian cancer	PFS based	88	65	CC,CG+GG
Saldivar	2007	USA	18	epithelial ovarian cancer	TFI	67	40	CC,CG,GG
Sullivan	2014	Spain	19	non-small cell lung cancer^†^	RECIST	58	16	CC,CG,GG
Sullivan	2014	Spain	19	non-small cell lung cancer^‡^	RECIST	31	56	CC+CG,GG
Yu	2007	China	20	non-small cell lung cancer	WHO	41	60	CC,CG,GG
Zhang	2013	China	9	non-small cell lung cancer	EORCT	137	314	CC,CG,GG

PFS based: Progression free survival based.

TRG: tumor regression grade.

EORTC: European Organization for Research on Treatment of Cancer.

NCCN: The National Comprehensive Cancer Network.

TFI: treatment-free interval after initial chemotherapy.

RECIST: response evaluation criteria in solid tumors.

^†^TNM stage III group in Sullivan’s study (2014).

^‡^TNM stage IV group in Sullivan’s study (2014).

### Meta-analysis results

**Figure 2:**
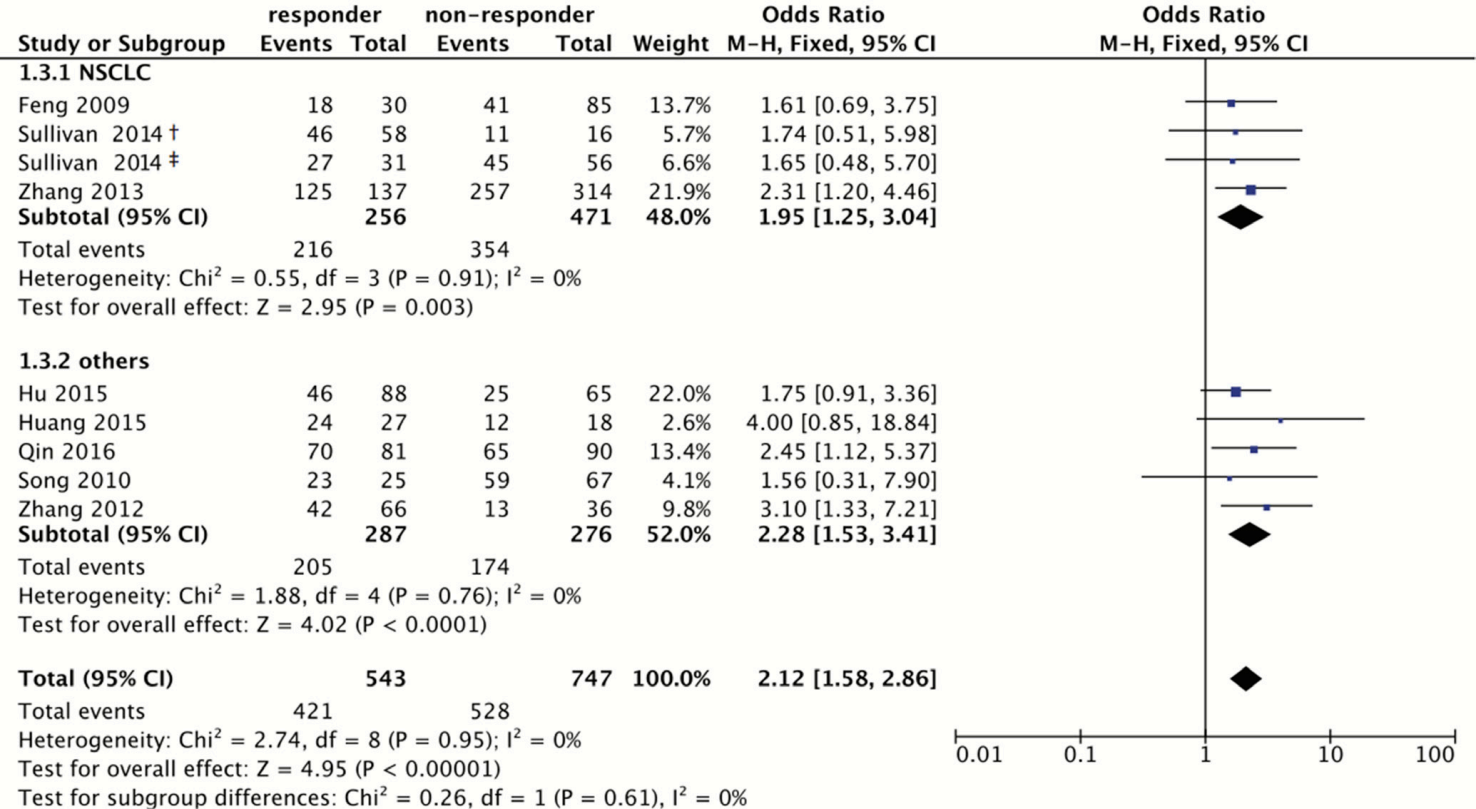
Forest plot for associations of the *ERCC5* rs1047768 polymorphism with the sensitivity to platinum-based chemotherapy Odds ratios with 95% confidence intervals were calculated under the dominant model (CC+CT vs. TT). Abbreviations: OR, odds ratio; 95% CI, 95% confidence interval; df, degree of freedom; NSCLC, non-small cell lung cancer. The diamonds reflect the pooled ORs and 95% CIs of the overall population and the individual subgroups. ^†^marks the TNM stage III group in Sullivan’s study (2014), and ^‡^indicates the TNM stage IV group in Sullivan’s study (2014). Each involved study was represented by a violet square and a black horizontal line, representing the point estimate of OR and the corresponding 95% CI, respectively. The area of each square is proportional to the weight of each study involved. The null effect is marked by a solid vertical line (labelled 1 on the x-axis). If the 95% CI does not cross 1.0 (null effect), it indicates significant effects (*P* ≤ 0.05). Otherwise, it means the effect estimate was non-significant (*P* > 0.05).

When integrating all eligible studies, 622 good responders and 884 poor responders to platinum-based chemotherapy were pooled into the meta-analysis of the rs1047768 polymorphism. The pooled odds ratio (OR) and 95% confidence interval (CI) revealed the C allele carriers could be more sensitive to platinum-based chemotherapy treatment (Homozygote model: OR = 2.86, 95% CI: 1.93–4.23; Heterozygote model: OR = 1.74, 95% CI: 1.21–2.51; Dominant model: OR = 2.12, 95% CI: 1.58–2.86; Recessive model: OR = 2.27, 95% CI: 1.54–3.33; Figure 2 and Table 2).

**Table 2:** Association between the *ERCC5* rs1047768 polymorphism and sensitivity to platinum-based chemotherapy

Comparison	Homozygote model (CC vs. TT)			Heterozygote model (CT vs. TT)			Dominant model (CC+CT vs. TT)			Recessive model (CC vs. CT+TT)		
	OR (95% CI)	*P*	*P*_h_	OR (95% CI)	*P*	*P*_h_	OR (95% CI)	*P*	*P*_h_	OR (95% CI)	*P*	*P*_h_
Overall	2.86 (1.93,4.23)	0.00	0.47	1.74 (1.21,2.51)	0.00	0.97	2.12 (1.58,2.86)	0.00	0.95	*2.27 (1.54,3.33)*	*0.00*	*0.04*
Cancer Type												
NSCLC	2.21 (1.34,3.65)	0.00	0.68	1.79 (1.10,2.89)	0.02	0.86	1.95 (1.25,3.04)	0.00	0.91	1.66 (1.23,2.23)	0.00	0.15
Others	4.25 (2.25,8.02)	0.00	0.47	1.68 (0.96,2.95)	0.07	0.78	2.28 (1.53,3.41)	0.00	0.76	3.31 (2.14,5.10)	0.00	0.29
Country												
China	3.30 (2.14,5.09)	0.00	0.60	1.69 (1.14,2.51)	0.01	0.89	2.18 (1.59,3.00)	0.00	0.87	2.29 (1.76,2.98)	0.00	0.17
Others	1.31 (0.50,3.47)	0.58	0.92	2.09 (0.80,5.47)	0.13	0.84	1.69 (0.70,4.07)	0.24	0.95	1.39 (0.46,4.24)	0.56	0.05
Response Criteria												
RECIST	2.08 (0.99,4.36)	0.05	0.23	1.72 (0.80,3.67)	0.16	0.93	1.97 (0.99,3.94)	0.05	0.80	*2.25 (1.20,4.23)*	*0.01*	*0.02*
Others	3.21 (2.02,5.10)	0.00	0.72	1.75 (1.15,2.66)	0.01	0.70	2.16 (1.55,3.00)	0.00	0.79	2.05 (1.50,2.81)	0.00	0.21

P_h_: *P* values for heterogeneity.

RECIST: Response Evaluation Criteria In Solid Tumors.

NSCLC: non-small cell lung cancer.

**Figure 3:**
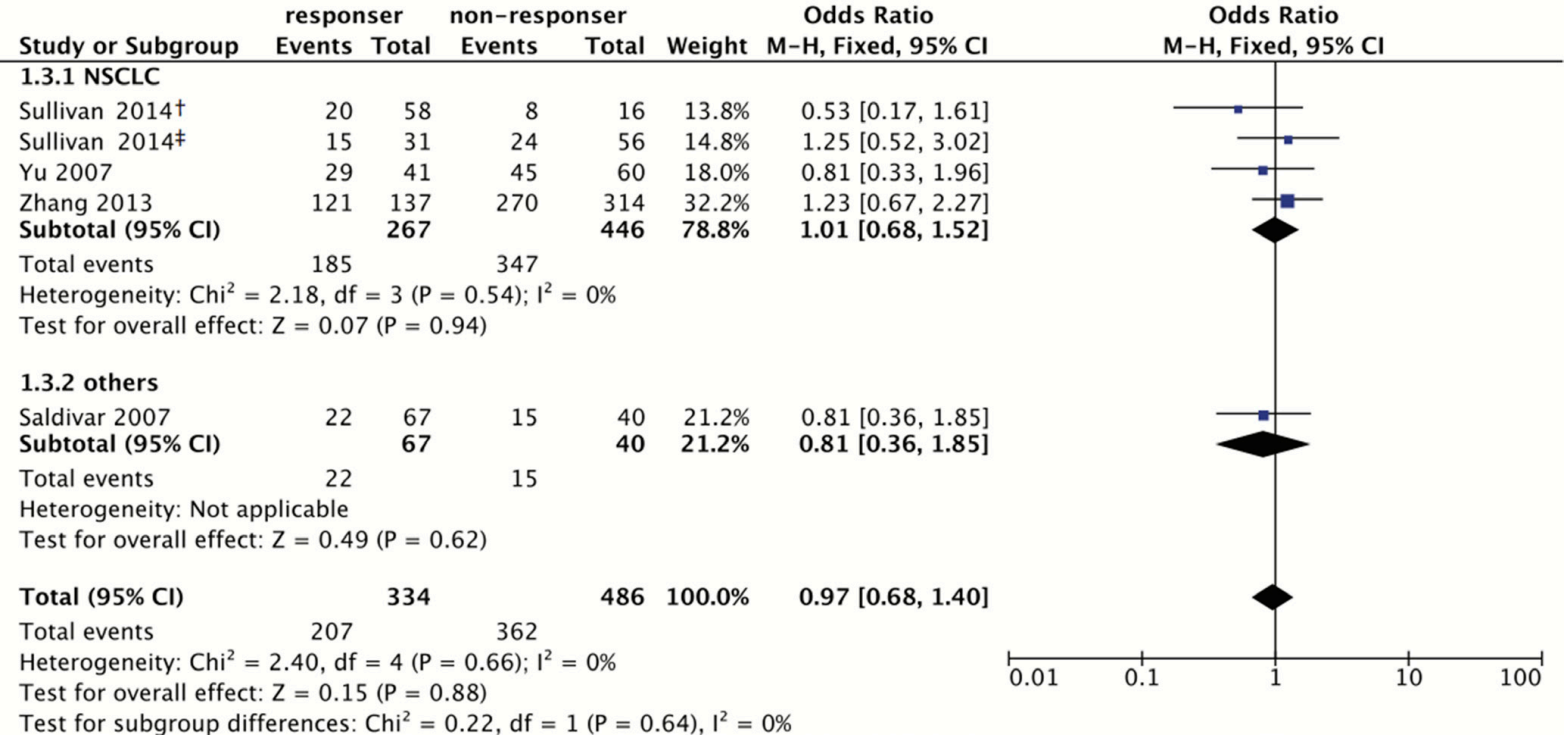
Forest plot for associations of the *ERCC5* rs17655 polymorphism with the sensitivity to platinum-based chemotherapy Odds ratios with 95% confidence intervals were calculated under the dominant model (CC+CG vs. GG). Abbreviation: OR, odds ratio; 95% CI, 95% confidence interval; df, degree of freedom; NSCLC, non-small cell lung cancer. The diamonds reflect the pooled ORs and 95% CIs of the overall population and the individual subgroups. ^†^marks the TNM stage III group in Sullivan’s study (2014), and ^‡^indicates the TNM stage IV group in Sullivan’s study (2014). Each involved study was represented by a violet square and a black horizontal line, representing the point estimate of OR and the corresponding 95% CI, respectively. The area of each square is proportional to the weight of each study involved. The null effect is marked by a solid vertical line (labelled 1 on the x-axis). If the 95% CI does not cross 1.0 (null effect), it indicates significant effects (*P* ≤ 0.05). Otherwise, it means the effect estimate was non-significant (*P* > 0.05).

For rs17655, 422 patients were sensitive, and 551 were non-sensitive. The same analysis was employed, and showed the association is not significant (Homozygote model: OR = 1.03, 95% CI: 0.63–1.67; Heterozygote model: OR = 0.90, 95% CI: 0.58–1.39; Dominant model: OR = 0.97, 95% CI: 0.68–1.40; Recessive model: OR = 1.04, 95% CI: 0.76–1.44; Figure 3 and Table 3).

**Table 3:** Association between the *ERCC5* rs17655 polymorphism and sensitivity to platinum-based chemotherapy

Comparison	Homozygote model (CC vs. GG)			Heterozygote model (CG vs. GG)			Dominant model (CC+CG vs. GG)			Recessive model (CC vs. CG+GG)		
	OR (95% CI)	*P*	*P*_h_	OR (95% CI)	*P*	*P*_h_	OR (95% CI)	*P*	*P*_h_	OR (95% CI)	*P*	*P*_h_
Overall	1.03 (0.63,1.67)	0.92	0.54	0.90 (0.58,1.39)	0.64	0.65	0.97 (0.68,1.40)	0.88	0.66	1.04 (0.76,1.44)	0.79	0.75
Cancer type												
NSCLC	1.11 (0.67,1.85)	0.69	0.63	0.89 (0.54,1.48)	0.66	0.44	1.01 (0.68,1.52)	0.94	0.54	1.12 (0.79,1.60)	0.52	0.83
Others	0.37 (0.06,2.37)	0.29	NA	0.93 (0.39,2.20)	0.86	NA	0.81 (0.36,1.85)	0.62	NA	0.76 (0.37,1.58)	0.47	0.41
Country												
China	1.17 (0.69,2.00)	0.55	0.63	0.99 (0.57,1.73)	0.98	0.38	1.08 (0.65,1.78)	0.76	0.44	1.10 (0.79,1.52)	0.59	0.82
Others	0.44 (0.12,1.59)	0.21	0.79	0.77 (0.38,1.56)	0.47	0.46	0.86 (0.51,1.46)	0.59	0.48	0.50 (0.15,1.74)	0.28	0.67
Response Criteria												
RECIST	0.53 (0.09,3.21)	0.49	NA	0.53 (0.16,1.77)	0.30	NA	0.90 (0.45,1.79)	0.77	0.23	0.66 (0.12,3.77)	0.64	NA
Others	1.07 (0.65,1.78)	0.78	0.45	0.97 (0.61,1.55)	0.91	0.68	1.00 (0.65,1.53)	1	0.63	1.06 (0.77,1.46)	0.73	0.65

*P*_h_: *P* values for heterogeneity.

RECIST: Response Evaluation Criteria In Solid Tumors.

NSCLC: non-small cell lung cancer.

### Subgroup analysis

Stratified analyses were conducted for the data of both the rs1047768 and rs17655 polymorphisms. Cancer type (NSCLC or others), country (China or others), and chemotherapy response evaluation criteria (response evaluation criteria in solid tumors (RECIST) or others) were the factors considered. ORs and 95% CIs were recalculated for every individual subgroup.

An association between the rs1047768 polymorphism and sensitivity to platinum-based chemotherapy could be detected in both the NSCLC and non-NSCLC subgroups. There was an association in the Chinese population, but not in the subgroup for other nations. When stratifying according to chemotherapy response evaluation criteria, there was an association to only the recessive model in the RECIST subgroup. However, as for the non-RECIST subgroup, all four comparing models showed significant associations. No subgroup correlations were found for the rs17655 polymorphism.

### Heterogeneity analysis

Heterogeneity was only identified in the recessive model of the *ERCC5* rs1047768 polymorphism. When stratified according to cancer type, heterogeneity was significantly relieved in both subgroups. However, neither country nor chemotherapy response evaluation criteria was identified as the contributing factor for between-study heterogeneity.

### Publication bias and sensitivity analysis

**Figure 4:**
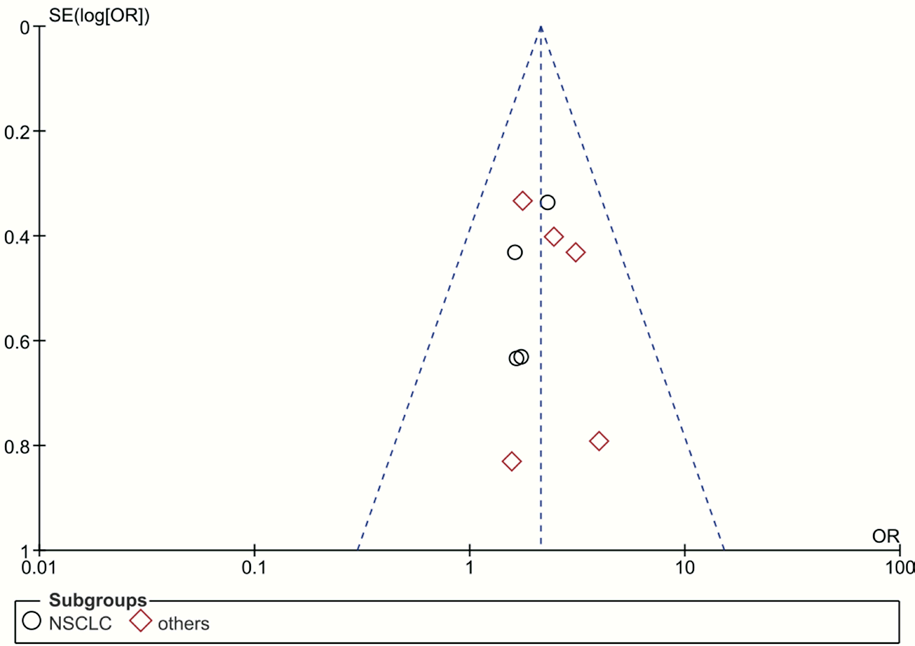
Funnel plot for publication bias of the rs1047768 polymorphism studies used in the dominant model (CC+CT vs. TT) Abbreviations: OR, odds ratio; SE, standard error; log[OR], natural logarithm of OR; NSCLC, non-small cell lung cancer. Each spot represents one single study and different plotting symbols distinguish different subgroups (cancer types). The black rings indicate NSCLC studies, while the red diamonds correspond to other cancer types. The vertical dash line denotes the overall OR estimate. The two oblique dash lines mark the triangular 95% confidence region based on the meta-analysis with the fixed-effect model.

**Figure 5:**
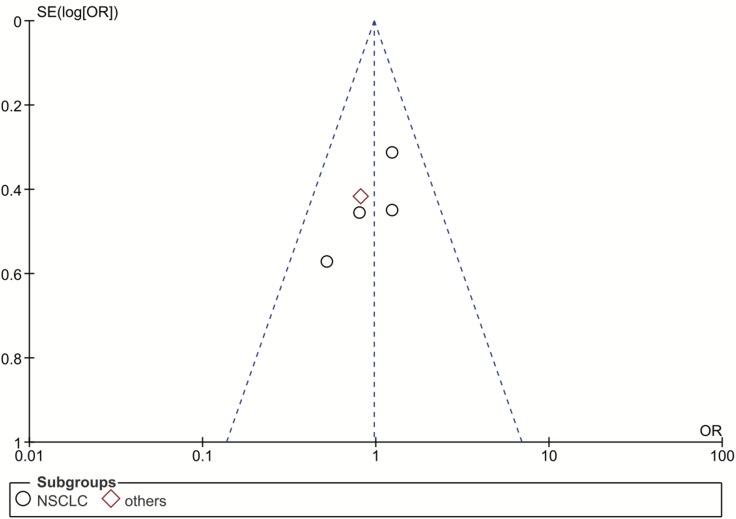
Funnel plot for publication bias of the rs17655 polymorphism studies used in the dominant model (CC+CG vs. GG) Abbreviations: OR, odds ratio; SE, standard error; log[OR], natural logarithm of OR; NSCLC, non-small cell lung cancer. Each spot represents one single study and different plotting symbols distinguish different subgroups (cancer types). The black rings indicate NSCLC studies, while the red diamonds correspond to other cancer types. The vertical dash line denotes the overall OR estimate. The two oblique dash lines mark the triangular 95% confidence region based on the meta-analysis with the fixed-effect model.

Our funnel plot analysis indicated a very small likelihood of publication bias in this study (Figure 4 and Figure 5). Our leave-one-out method showed that no single study could influence the pooled ORs and 95% CIs of the meta-analysis (data not shown).

## DISCUSSION

A predictive biomarker for sensitivity to platinum-based chemotherapy would increase the efficacy of personalized cancer treatment. The NER pathway promotes the repair for platinum-induced DNA damage [27], and genetic alterations in this pathway may consequently affect response to platinum chemotherapeutic agents. In the four genetic models, the pooled ORs and the corresponding 95% CIs of the *ERCC5* rs1047768 polymorphism indicated that the C allele may promote sensitivity to platinum-based chemotherapy. However, the rs17655 polymorphism showed no difference among different genotypes.

Stratified analyses based on cancer type, country, and chemotherapy response evaluation criteria were employed to detect subgroup difference. All subgroup results for the *ERCC5* rs17655 polymorphism showed no association with sensitivity to platinum-based chemotherapy. For the rs1047768 polymorphism, the C allele was associated with a significantly higher platinum sensitivity in both subgroups classified by cancer types. When stratified according to chemotherapy response evaluation criteria, the association in the RECIST subgroup was present only in the recessive model. However, large discrepancies existed between these two subgroups in the other three genetic comparison models. The stratified analysis by country revealed that significant association in the Chinese population, but not the other countries. Although we cannot exclude that we observed this difference due to the variation of genetic background among different ethnicities, it is also possible that the limited number for involved studies and samples from other nations led to this inconsistency.

The source of heterogeneity in any meta-analysis should be comprehensively investigated to avoid possible distortion. Heterogeneity was detected in the recessive model of the rs1047768 polymorphism. To screen out the source of heterogeneity, subgroup analyses according to different factors were conducted. When stratifying according to cancer type, neither the NSCLC nor non-NSCLC subgroup exhibited heterogeneity. This indicated that cancer type accompanied by different pathogenesis and some other underlying drug response mechanisms might be the confounding factor which account for the heterogeneity in this comparison.

Our meta-analysis had the following limitations. Other polymorphisms can influence ERCC5 mRNA or protein production (such as rs2296147, rs4150351, rs873601, and rs751402) [28–31], and they were not studied here. Their impacts on the sensitivity to platinum-based chemotherapy could not be assessed in this meta-analysis due to the lack of relevant case-control studies. Only publications in Chinese and English were selected. The number of non-Chinese studies, and the corresponding subgroup sample size, may have been too small. Clinical parameters, such as response evaluation criteria for chemotherapy, varied between different studies and the use of these parameters is also subject to the personal experience of the respective researchers, which may affect precision. Lastly, gender differences, lifestyle, and environmental effects were not taken into account.

The entire study workflow strictly adhered to the instructions of the Preferred Reporting Items for Systematic Reviews and Meta-Analysis (PRISMA) statement [32]. The robustness of our results was endorsed by both the sensitivity analysis and the publication bias analysis. These two merits can ensure the reliability of our results.

## MATERIALS AND METHODS

### Literature searching strategies

The official name or alias for the ERCC5 gene (ERCC5, ERCC5-201, COFS3, COFS3-201, ERCM2, UVDR, XPG, and XPGC), the terms for cancer (epithelioma, adenocarcinoma, osteosarcoma, carcinoma, and cancer), and the keywords standing for single nucleotide polymorphism (polymorphism, SNP, and variant) were utilized to form a boolean expression. This expression was systematically queried in Cochrane Library, Web of Science, and PubMed to collect potentially related studies published in English. All searches in these databases were finalized September 21, 2016.

To identify the possibly relevant studies published in Chinese, the same boolean expression was then used in the China National Knowledge Infrastructure (CNKI) database. Additional search queries for chemotherapy (platinum, Cisplatin, Carboplatin, and Oxaliplatin) were used to filter the resulting publications. The Chinese literature search was finalized October 4, 2016.

### Study selection

All included studies met the following selection criteria: (1) from peer-reviewed journals published in English or Chinese language; (2) unrelated case-control studies evaluating the association between the *ERCC5* polymorphisms (rs1047768 and rs17655), and the sensitivity to the platinum-based chemotherapy for cancers; (3) genotype frequency data available was sufficient to build at least one genetic comparison model; (4) all values relating to *ERCC5* polymorphism genotype frequencies were correct and not contradictory; (5) contained definitive chemotherapy response criteria. When two or more publications shared the same case and control samples, only the earliest study was included. Relevant publications without available data, even after email requests to their first/corresponding authors, were excluded. Four investigators participated in the selection, and another reviewer did a comprehensive inspection of the included studies.

### Data extraction

Information including first author’s family name, year of publication, country, cancer type, genotype frequencies for the *ERCC5* rs1047768 and rs17655 polymorphisms, and tumor response criteria were extracted from the identified studies by four investigators. Studies were re-checked to further confirm that they evaluated the association between the *ERCC5* rs1047768 and rs17655 polymorphisms and the sensitivity to platinum-based therapies.

### Statistics analysis

All statistics analyses in this meta-analysis were fulfilled using Review Manager software (Version 5.3. The Nordic Cochrane Centre, The Cochrane Collaboration, Copenhagen, Denmark).

The extracted data for both the *ERCC5* rs1047768 and rs17655 polymorphisms from all included studies were tested using the homozygote (rs1047768: CC vs. TT; rs17655: CC vs. GG), heterozygote (rs1047768: TC vs. TT; rs17655: GC vs. GG), dominant (rs1047768: CC+TC vs. TT; rs17655: CC + GC vs. GG), and recessive (rs1047768: CC vs. TT + TC; rs17655: CC vs. GG + GC) genetic models. The ORs with 95% CIs were calculated to measure the association between these *ERCC5* polymorphisms and platinum-based chemotherapy sensitivity. Heterogeneity was evaluated to make the choice between the random-effect (DerSimonian-Laird algorithm) [33] and the fixed-effect (Mantel-Haenszel algorithm) [34] models. When significant heterogeneity existed (*P* < 0.10), the random-effect model was applied. Otherwise, fixed-effect model was used.

To evaluate the differences among subgroups, stratified comparisons between cancer types (NSCLC or others), countries (China or others), and chemotherapy response criteria (RECIST or others) were conducted. Publication bias was visually assessed via the funnel plot generated by Review Manager. To ensure stability of the results, the one-by-one sensitivity analysis with replacement was used to recalculate the ORs and 95% CIs on the remaining studies.

## CONCLUSIONS

Our meta-analysis showed that C allele carriers of the *ERCC5* rs1047768 polymorphism are more sensitive to platinum-based chemotherapy, especially for Chinese patients. However, the *ERCC5* rs17655 polymorphism is not associated with sensitivity to platinating agents.

## References

[1] Shen DW, Pouliot LM, Hall MD, Gottesman MM (2012). Cisplatin Resistance: A Cellular Self-Defense Mechanism Resulting from Multiple Epigenetic and Genetic Changes. Pharmacol Rev.

[2] Reed E (1998). Platinum-DNA adduct, nucleotide excision repair and platinum based anti-cancer chemotherapy. Cancer Treat Rev.

[3] Wei Q, Frazier ML, Levin B (2000). DNA Repair: a Double-Edged Sword. J Natl Cancer Inst.

[4] Rosell R, Taron M, Barnadas A, Scagliotti G, Sarries C, Roig B (2003). Nucleotide excision repair pathways involved in Cisplatin resistance in non-small-cell lung cancer. Cancer Control.

[5] Chen J, Wang Z, Zou T, Cui J, Yin J, Zheng W, Jiang W, Zhou H, Liu Z (2016). Pharmacogenomics of platinum-based chemotherapy response in NSCLC: a genotyping study and a pooled analysis. Oncotarget.

[6] Du Y, Su T, Zhao L, Tan X, Chang W, Zhang H, Cao G (2014). Associations of Polymorphisms in DNA Repair Genes and MDR1 Gene with Chemotherapy Response and Survival of Non-Small Cell Lung Cancer. PLoS One.

[7] Shen X, Lu F, Wu Y, Zhao L, Lin Z (2013). XRCC3 Thr241Met Polymorphism and Clinical Outcomes of NSCLC Patients Receiving Platinum-Based Chemotherapy: A Systematic Review and Meta-Analysis. PLoS One.

[8] Negandhi AA, Hyde A, Dicks E, Pollett W, Younghusband BH, Parfrey P, Green RC, Savas S (2013). MTHFR Glu429Ala and ERCC5 His46His Polymorphisms Are Associated with Prognosis in Colorectal Cancer Patients: Analysis of Two Independent Cohorts from Newfoundland. PLoS One.

[9] Zhang T, Sun J, Lv M, Zhang L, Wang X, Ren JC, Wang B (2013). XPG is predictive gene of clinical outcome in advanced non-small-cell lung cancer with platinum drug therapy. Asian Pac J Cancer Prev.

[10] Kweekel DM, Antonini NF, Nortier JWR, Punt CJA, Gelderblom H, Guchelaar HJ (2009). Explorative study to identify novel candidate genes related to oxaliplatin efficacy and toxicity using a DNA repair array. Br J Cancer.

[11] Li Y, Liu Z, Liu H, Wang LE, Onodera H, Suzuki A, Suzuki K, Wadhwa R, Elimova E, Sudo K, Shiozaki H, Estrella J, Lee JS (2014). Potentially functional variants in the core nucleotide excision repair genes predict survival in Japanese gastric cancer patients. Carcinogenesis.

[12] Schrama D, Scherer D, Schneider M, Zapatka M, Brocker EB, Schadendorf D, Ugurel S, Kumar R, Becker JC (2011). ERCC5 p.Asp1104His and ERCC2 p.Lys751Gln polymorphisms are independent prognostic factors for the clinical course of melanoma. J Invest Dermatol.

[13] Ito S, Kuraoka I, Chymkowitch P, Compe E, Takedachi A, Ishigami C, Coin F, Egly JM, Tanaka K (2007). XPG stabilizes TFIIH, allowing transactivation of nuclear receptors: implications for Cockayne syndrome in XP-G/CS patients. Mol Cell.

[14] Caiola E, Porcu L, Fruscio R, Giuliani D, Milani R, Torri V, Broggini M, Marabese M (2013). DNA-damage response gene polymorphisms and therapeutic outcomes in ovarian cancer. Pharmacogenomics J.

[15] Feng JF, Sun XC, Sun N, Qin SK, Li F, Cheng HY, Chen BO, Cao YD, Ma J, Cheng L, Lu ZH, Ji JZ, Zhou YF (2009). XPA A23G polymorphism is associated with the elevated response to platinum-based chemotherapy in advanced non-small cell lung cancer. Acta Biochim Biophys Sin.

[16] Monzo M, Moreno I, Navarro A, Ibeas R, Artells R, Gel B, Martinez F, Moreno J, Hernandez R, Navarro-Vigo M (2007). Single nucleotide polymorphisms in nucleotide excision repair genes XPA, XPD, XPG and ERCC1 in advanced colorectal cancer patients treated with first-line oxaliplatin/fluoropyrimidine. Oncology.

[17] Qin YZ, Tang F, Chen JS, Lin Y, Lai H (2016). DNA repair gene XPG C46T polymorphism predicts response to platinum-based neoadjuvant chemoradiotherapy and prognosis in clinical stage II/III rectal cancer patients. Int J Clin Exp Pathol.

[18] Saldivar JS, Lu KH, Liang D, Gu J, Huang M, Vlastos AT, Follen M, Wu XF (2007). Moving toward individualized therapy based on NER polymorphisms that predict platinum sensitivity in ovarian cancer patients. Gynecol Oncol.

[19] Sullivan I, Salazar J, Majem M, Pallares C, del Rio E, Paez D, Baiget M, Barnadas A (2014). Pharmacogenetics of the DNA repair pathways in advanced non-small cell lung cancer patients treated with platinum-based chemotherapy. Cancer Letters.

[20] Yu QZ, Han JX, Pan JH, Shen LJ, Wu JM, Huang HN (2007). The Relationship between the Polymorphisms of Gene XPG and MDR1 and the Responsiveness of Advanced Non-small Cell Lung Cancer to Platinum-based Chemotherapy. Practical J Cancer.

[21] Lv HY, Li QC, Wei HJ, Xiang JY, Yao RY, Liang J (2012). Relationship between GSTP1 and XPG genetic polymorphisms and survival of platinum-based chemotherapy in advanced non-small cell ung cancer patients. China Oncol.

[22] Song SA, Liang J, Jiang T, Jiang J, Sun YY, Li QF (2010). Correlation between XPG gene polymophism and therapeutic effect of Oxaliplatin for advanced gastric cancer. Medical Journal of Qilu.

[23] Zhang W, Wu FX, Wang Q, Li DJ, Li L (2012). The relation between XPG genetic polymorphism and the sensitivity to platinum-based chemotherapy in patients with ovarian cancer. Chinese J Prac Gynecol Obstet.

[24] Hu P, Kang S, Zhou RM, Wang N, Qi BL, Li Y (2015). The association between the polymorphisms of XPG tagSNPs and the clinical outcomes of epithelial ovarian cancer patients treated with platinum-based chemotherapy. Chinese J Prac Gynecol Obstet.

[25] Jia XF, Liang J, Lv HY, Yao RY, Zhou F, Han Y (2011). Relationship between XPA and XPG polymorphisms and platinum-based chemotherapy outcomes in advanced non-small cell lung cancer. Prog Mod Biomed.

[26] Huang WY, Tang F, Sun S, Cao CP, Wang B (2015). Relationship between curative effect of neoadjuvant chemoradiotherapy on rectal cancer and XPG C46T gene polymorphisms in rectal cancer tissues. Prog Mod Biomed.

[27] Bonanno L, Favaretto A, Rosell R (2014). Platinum drugs and DNA repair mechanisms in lung cancer. Anticancer Res.

[28] Wang B, Xu Q, Yang HW, Sun LP, Yuan Y (2016). The association of six polymorphisms of five genes involved in three steps of nucleotide excision repair pathways with hepatocellular cancer risk. Oncotarget.

[29] Chen Y, Zhou F, Shen D, He X, Zhang Y, Xu L, Xia Q, Liang S, Song Y, Zuo Y (2016). ERCC5 single nucleotide polymorphism (rs2296147) predicts the risk of acute radiation pneumonitis in lung cancer patients undergoing radiotherapy. Int J Clin Exp Pathol.

[30] Ma H, Yu H, Liu Z, Wang LE, Sturgis EM, Wei Q (2012). Polymorphisms of XPG/ERCC5 and risk of squamous cell carcinoma of the head and neck. Pharmacogenet Genomics.

[31] Somers J, Wilson LA, Kilday JP, Horvilleur E, Cannell IG, Poyry TA, Cobbold LC, Kondrashov A, Knight JR, Puget S, Grill J, Grundy RG, Bushell M (2015). A common polymorphism in the 5′ UTR of ERCC5 creates an upstream ORF that confers resistance to platinum-based chemotherapy. Genes Dev.

[32] Moher D, Liberati A, Tetzlaff J, Altman DG, PRISMA Group (2009). Preferred Reporting Items for Systematic Reviews and Meta-Analyses: The PRISMA Statement. PLoS Med.

[33] DerSimonian R, Laird N (1986). Meta-analysis in clinical trials. Control Clin Trials.

[34] Mantel N, Haenszel W (1959). Statistical aspects of the analysis of data from retrospective studies of disease. J Natl Cancer Inst.

